# Therapeutic Efficacy of Stem Cell-based Therapy in Peripheral Arterial Disease: A Meta-Analysis

**DOI:** 10.1371/journal.pone.0125032

**Published:** 2015-04-29

**Authors:** Yumeng Liu, Yunyun Xu, Fang Fang, Jianting Zhang, Liang Guo, Zhen Weng

**Affiliations:** 1 Department of Radiology, the First Affiliated Hospital of Soochow University, Suzhou, Jiangsu, China; 2 Institute of Pediatrics, Children’s Hospital Affiliated to Soochow University, Suzhou, Jiangsu, China; 3 Key Laboratory of Nano-Bio Interface, Division of Nanobiomedicine and i-Lab, Suzhou Institute of Nano-Tech and Nano-Bionics, Chinese Academy of Sciences, Suzhou, Jiangsu, China; Bristol Heart Institute, University of Bristol, UNITED KINGDOM

## Abstract

Several cell-based therapies for peripheral arterial disease (PAD) have been studied in multiple clinical trials; however, the outcome of this treatment remains controversial. The aim of this study was to perform a meta-analysis of the clinical trials on stem cell-based therapy after PAD. We searched for clinical trials that investigated the effect of stem cell-based therapy on patients with PAD and were published between January 2000 and October 2014. The outcomes of interest comprised all-cause mortality, amputation rate, ulcer healing, and ankle-brachial index (ABI). In addition, pooled odds ratios (ORs) and 95% confidence intervals (CIs) were calculated to assess the safety and efficacy of the stem cell-based therapies for PAD. Thirteen studies were retrieved from 261 citations for the analysis, and in total, 527 patients (mean age: 64.2 years; median follow up: 6 months) were included in the analysis. After synthesizing data, the meta-analysis showed significant improvement in the amputation rate (OR=0.33, 95%CI=0.22-0.51; P<0.001), ulcer healing (OR=6.11, 95%CI=3.04-12.28; P<0.001), and ABI (SMD=0.65, 95%CI=0.33-0.97; P<0.001) for the stem cell-based therapy group compared with the controls. Moreover, significant improvement in the amputation rate, ulcer healing, and ABI were also found based on the time point and stem cell source. In addition, no significant difference was found in the all-cause mortality (OR=0.80, 95%CI=0.39-1.641; P=0.546) between the stem cell-based therapy and control groups. Therefore, according to the results of our meta-analysis, stem cell-based therapy is safe and shows a beneficial outcome for patients with PAD, especially in the short term.

## Introduction

Peripheral arterial disease (PAD), which manifestsin 10–20% of people over 65 years of age, is a common circulatory disease in which narrowed arteries reduce blood flow in the limbs (for most conditions) [1–3]. A large burden of morbidity and mortality can be found in patients with PAD due to its close relationship with coronary artery disease (CAD) and cerebrovascular disease (CVD) [4]. Therefore, the search for novel therapeutic approaches thatstimulate vascular regeneration and improve blood perfusion after PAD is now under active development.

Currently, several new therapeutic approaches, including exercise therapy, pharmacotherapy, and revascularization surgery, have been proposed by different research groups. In 2002, Tateishi-Yuyama *et al* concluded that transplantation of autologous bone marrow mononuclear cells (BMMNCs) was safe and effective for achieving therapeutic angiogenesis in patients with limb ischemia and could obtain better clinical outcomes after being injectedinto the involved limb. Since then, several cell-based therapies using bone marrow or mobilized peripheral blood haveproven that limb ischemia canbe improved after cell transplantation;however, controversiesregarding the safety and efficacy of cellular therapy remain because of the limited number of treated patients and variable procedures.

Here, we performed a meta-analysis focusing on PAD patients who hadbeen treated with an infusion of BMMNCs, bone marrow-derived mesenchymal stem cells (BMMSCs), granulocyte colony-stimulating factormobilized peripheral blood mononuclear cells (G-CSF PBMCs), or peripheral blood-derivedstemcells(VesCell). Our data provides a comparison of cell-based therapy and placebo in patients with PAD.

## Materials and Methods

### Literature search, selection, and data collection

Papers regarding stem cell-based therapy in patients with PAD that were published onPubMed and Web of Science between January 2000 and October 2014 were included in this meta-analysis. The following search terms were used: stem cells, progenitor cells, mononuclear cells, adipose tissue-derived regenerative cells, MSCs, vascular-derived stem cells, bone marrow, vascular stromal fraction, adipose stem cells, mesenchymal-like stem cells, peripheral artery disease, peripheral arterial disease, PAD, claudication, limb ischaemia, and limb ischemia. Studies that met the following criteria were included: (1) was a full-text, English-written study; (2) was a randomized trial or observational studywithanappropriate control group that received a sham injection; (3) included patients with established PAD, which was diagnosed based onthe presence of stable intermittent claudication and/or an ankle-brachial index (ABI) ≤0.9; (4) usedstem cells that were administered via intramuscular injection or intra-arterial injection; (5) included atotal number of enrolled patients that exceeded 10; (6) usedstem cells derived from adipose tissue, bone marrow, or mobilized peripheral blood; and (7) was given in an allogeneic or autologous setting.

Data abstraction and analysis was performed by 3 different researchers (Z.W., Y.L., Y.X.) and reported on standardized forms. Amputation, ulcer healing, ABI and all-cause mortality,as well as clinical outcome,were assessed as outcome measures. Additional subgroup analyses were performed in an attempt to gain more insight into the parameters or conditions that might improve outcome in the future. The subgroup analyses that were conducted included a follow-up duration of 3 months, 6 months, or12 months or longer as well asdifferent types of bone marrow-derived cells and mobilized peripheral blood-derived cells.

### Quality assessment

According to aprevious study, the Cochrane Collaboration’s tool [5], which is used to assess the risk of bias, was used to assess the methodological quality of the included studies. Seven items, including adequacy of randomization, allocation concealment, blinding (participants/personnel and outcome assessment), completeness of outcome data, selective reporting and the presence of any other bias, were evaluated.

### Data analysis

For the meta-analysis, pooled odds ratios (ORs) and 95% confidence intervals (CIs) were calculated using a fixed effects model or random effects model. The chosen model was based on the results of a heterogeneity test, which employed a previously described,χ^2^-based Q-test [6]. If the Q-test reported a p value greater than 0.1, afixedeffects model was usedaccording to the Mantel-Haenszel method;otherwise,arandom effects model was usedaccording to the DerSimonian and Laird model.

Publication bias was tested using Begg’s funnel plot and Egger’s test [7]. If the funnel plot was asymmetric and Egger’s test reported a p value less than 0.05, a publication bias probably existed.

Here, we performed all of the analyses using the Stata version 12.0 software (Stata Corporation, College Station, Texas, USA).

## Results

### Search results and study quality

**Fig 1:**
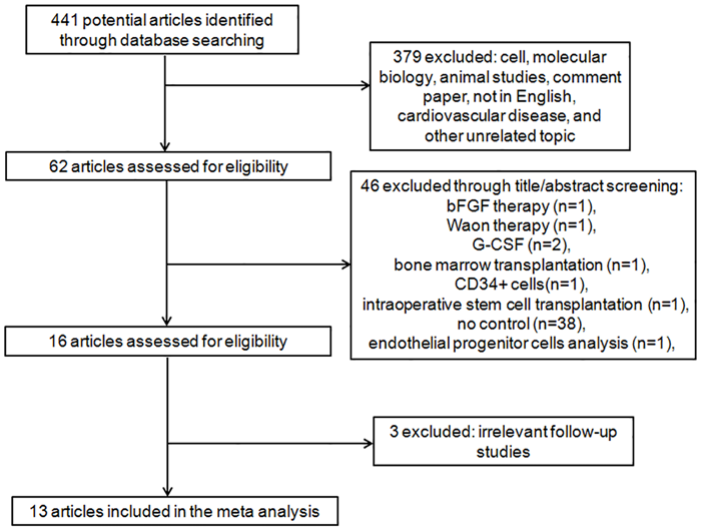
Flow chart of study inclusion.

The final search, which took place on October 31, 2014, resulted in 441 articles. A majority of the articles were excluded due to thetype of study subjects wasaboutcardiovascular disease or an unrelated topic or the useof animal studiesorif the article was a duplicated or review or commentaryarticle, resulting in a total of 62 included articles. After studies usinganother therapy or lacking the appropriate controls were omitted, 16 articles remained (S2 Table). Finally, 13 articles [8–20] were included in this meta-analysis, comprising 527 patients, 275 of whom were treated with a stem cell-based therapy. The review process is depicted in Fig 1 following previously published reporting recommendations [21]. Of the studies, 5 used mobilized peripheral blood mononuclear cells, 7 used bone marrow-derived cells,and1 used both mobilized peripheral blood mononuclear cells (PBMCs) and bone marrow-derived cells.

**Fig 2:**
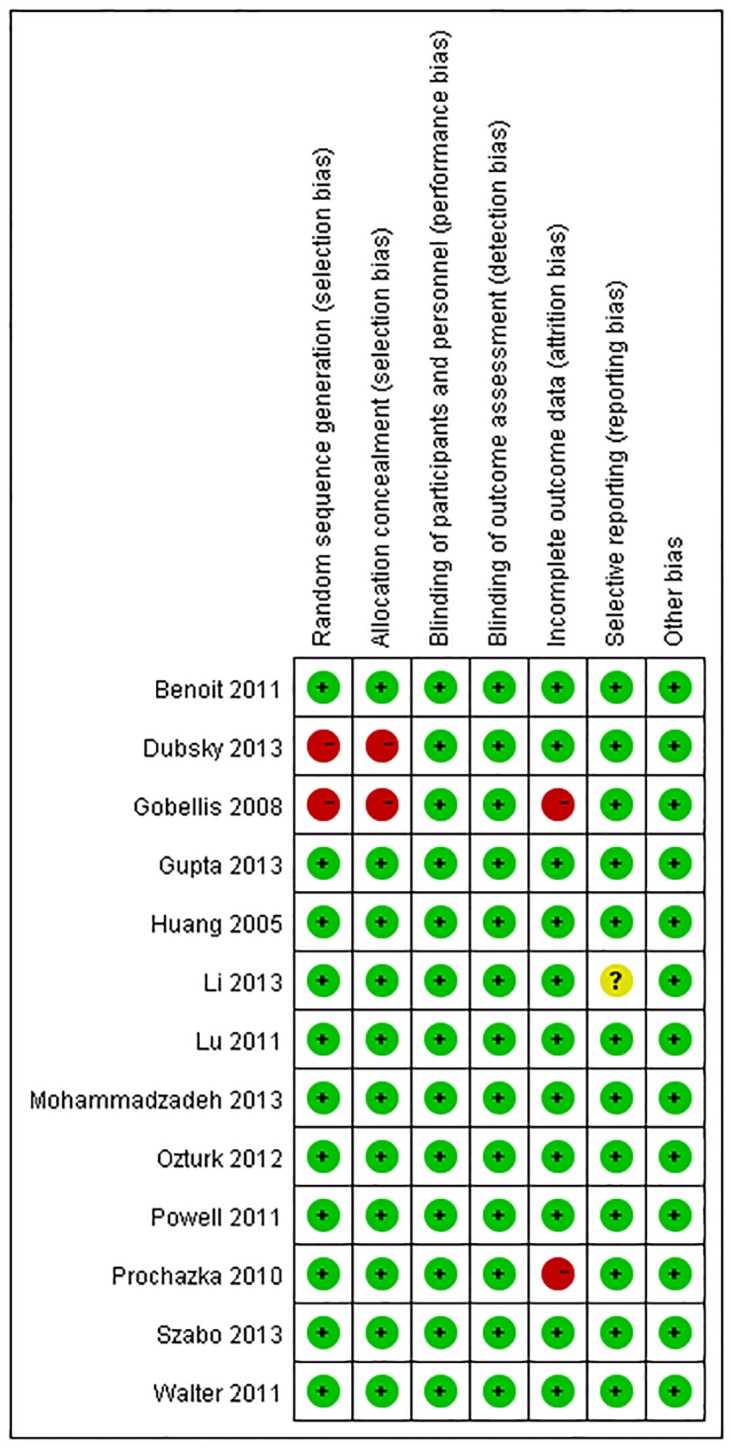
Risk of bias summary.

The risk of publication bias was summarized (Fig 2), and a low risk of bias was identified in most of the studies, except for the studies conducted by Gobellis *etal* [9] and Dubsky *et al* [10].

### Study Characteristics

The average number of participated patients per study was 40 ± 21, whereas the median was 40 patients (range, 19–96). A 1:1 randomization scheme was used in most studies, and the median follow-up duration forall of the studies was 6 months (range, 3–24 months). Granulocyte colony-stimulating factor-mobilized PBMCs (G-CSF PBMCs) as well as bone marrow-derived mononuclear cells (BMMNCs) were administered in 4 of the studies, bone marrow mesenchymal stem cells (BMMSCs) were administered in 3 of the studies, tissue repair cells were administered in 1 study, and VesCellwere administered in 1 study. The safety endpoint, efficacy endpoints, and angiogenesis modality for each individual study arelisted in Table 1.

**Table 1:** Main feature of included studies.

Study (Year)	Patients enrolled (Patients at follow up)	Cell type	Design	Follow up time points	Safety endpoint	Efficacy endpoint
**Huang et al (2005)**	28 (28)	G-CSF PBMCs	RCT	3 months	Death	Amputation, Ulcer healing, ABI
**Cobellis et al (2008)**	19(14)	Bone marrow	Nonrandom, controlled	6, 12 months	NR	Amputation, ABI
**Prochazka et al (2010)**	96(79)	BMMSCs	RCT	90 or 120 days	Death	Amputation
**Benoit et al (2011)**	48 (48)	Bone marrow aspirate Concentrate	RCT	1, 4, 8, 12, 26 weeks	Death	Amputation
**Lu et al (2011)**	41 (37)	BMMSCs, BMMNCs	RCT	24 weeks	Death	Amputation, Ulcer healing
**Powell et al (2011)**	46(46)	Tissue-repair cells	RCT	6 months, 12 months	Death	Amputation, Ulcer healing
**Walter et al (2011)**	40 (33)	BMMNCs	RCT	3 months	Death	Amputation, ABI
**Ozturk et al (2012)**	40 (40)	G-CSF PBMCs	RCT	12 weeks	Death	Amputation, Ulcer healing,ABI
**Mohammadzadeh et al (2013)**	21(21)	G-CSF PBMCs	RCT	3 months	Death	Amputation, Ulcer healing, ABI
**Dubsky et al (2013)**	50 (47)	BMMNCsG-CSF PBMCs	Non-random, controlled	6 months	Death	Amputation, Ulcer healing
**Li et al (2013)**	58 (58)	BMMNCs	RCT	6 months	Death	Amputation, Ulcer healing
**Gupta et al (2013)**	20 (19)	BMMSCs	RCT	1, 6 months	Death	Amputation,Ulcer healing, ABI
**Szabo et al (2013)**	20 (18)	VesCell	RCT	1, 3 months and 2 years	Death	Amputation, Ulcer healing

G-CSF: Granulocyte colony-stimulating factor; PBMCs: peripheral blood mononuclear cells; RCT: random controlled trial; ABI: ankle-brachial pressure; NR: not reported; BMMNCs: bone marrow-derived mononuclear cells; BMMSCs: bone marrow-derived mesenchymal stem cells.

### Patient and procedural characteristics

The mean age of the patientsintheincluded studies ranged from 44.9 to 71.4 years, and male patients dominated in all of the studies. The severity of disease was judged by Fontaine III-IV, Rutherford 4–6, ABI, and transcutaneous oxygen pressure (TcPO_2_) in most of the studies (11/13, 84.6%); and diabetes mellitus (DM) and hypertension werethe most commoncomorbiditiesfoundin the reported studies. The data on the days of infusion, number of injected cells, the number of CD34^+^ cells and the injected volume are listed in Table 2. The routes of cell administration were intramuscular and intra-arterial injection.

**Table 2:** Patient and procedural characteristics of the included studies.

Study	Mean age	Male (n, %)	Severity of disease	Smoking (n, %)	DM (n, %)	HP (n, %)	HL (n, %)	Number of injected cells (10^8^)	Administration route
				NP	28(100.0%)	NP	NP		
**Cobellis et al**	65.8	12(63.2%)	Fontaine stage III- IV	NP	NP	12(52.6%)	NP	10	IA
**Prochazka et al**	65.0	76(81.3%)	Rutherford 4–6, Fontaine IV;ABI≤ 0.4; ASP≤50 mm Hg; TSP≤ 30 mm Hg;	41(42.7%)	90(93.8%)	84(87.5%)	66(68.8%)	Bone marrow concentrate	IM
**Benoit et al**	69.5	32(66.7%)	Rutherford 4–5; ABI<0.4; TBI<0.4; TcPO_2_< 20 mm Hg	NP	24(50.0%)	NP	NP	NP	IM
**Lu et al**	64.4	29(72.5%)	Rutherford 4–6;	20(50.0%)	20(50.0%)	34(91.9%)	32(86.5%)	1.53 BMMNCs	IA
**Powell et al**	67.9	33(71.7%)	TSP≤50 mm Hg, ASP≤ 70 mm Hg	39(84.7%)	25(46.0%)	NP	NP	13.6	IM
**Walter et al**	64.4	29(72.5%)	Rutherford 4–6;	20(50.0%)	20(50.0%)	28(70%)	31(77.5%)	1.53 BMMNCs	IA
**Ozturk et al**	71.4	29 (72.5%)	Fontaine III-IV	NP	40(100%)	NP	NP	NP	IM
**Mohammadzadeh et al**	64.0	NP	Diabetic CLI with angioplasty failure	6(28.5%)	21(100%)	11(52.4%)	11(52.4%)	0.9–1.2	IM
**Dubsky et al**	62.4	41(82%)	Rutherford 4–6; ABI<0.6, TcPO_2_<30 mm Hg	33 (66.0%)	50(100.0%)	41(82.0%)	NP	NP	IM
**Li et al**	62	45(77.6%)	ABI<0.6, TSBP<30 mm Hg	47(81.0%)	25(43.1%)	48(81.4%)	NP	0.1	IM
**Gupta et al**	44.9	45(77.6%)	Rutherford 4–6; ABI ≤0.6, TcPO_2_≤60 mm Hg	20 (100%)	NP	NP	NP	0.2	IM
**Szabo et al**	61.8	13(65%)	Fontaine III-IV; ABI<0.45, TcPO_2_<40 mm Hg	3 (15%)	12(60%)	NP	5(25.0%)	0.66	IM

ABI: ankle-brachial index; TcPO_2_: transcutaneous oxygen pressure; IM: Intramuscular injection; TSBP: toe systolic blood pressure; NP: not provided; TSP: toe systolic pressure; ASP: ankle systolic pressure; DM:diabetes mellitus; HP: hypertension; HL:hyperlipidemia; IA: Intraarterial injection; TBI: toe-brachial index.

### Meta-analysis results

A standard meta-analysis was performed for the outcomes amputation (reported in 13 studies), ulcer healing (9 studies), ABI(6 studies) and all-cause mortality (12 studies), as sufficient data were available. For amputation, ulcer healing and ABI, the subgroup analysis results were presented based on the follow-up time and cell source. The detailed results of our meta-analysis are shown in S3 and S4 Tables.

**Fig 3:**
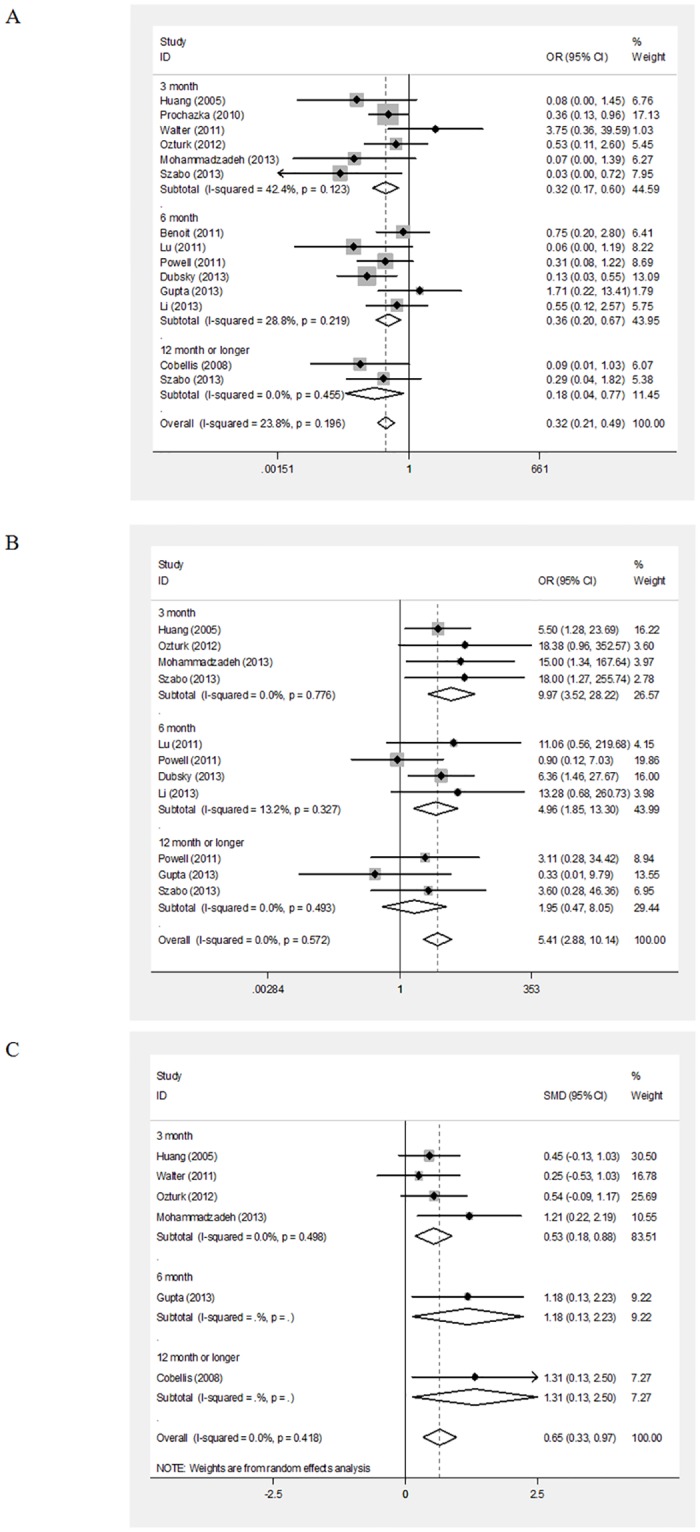
Forest plots of amputation (A), ulcer healing (B) and the ankle-brachial index (ABI) (C) over time. Amputation and ulcer healing were calculated using a fixedeffects model, while ABI was calculated using arandomeffects model. CI:confidence interval; OR:odds ratio.

**Fig 4:**
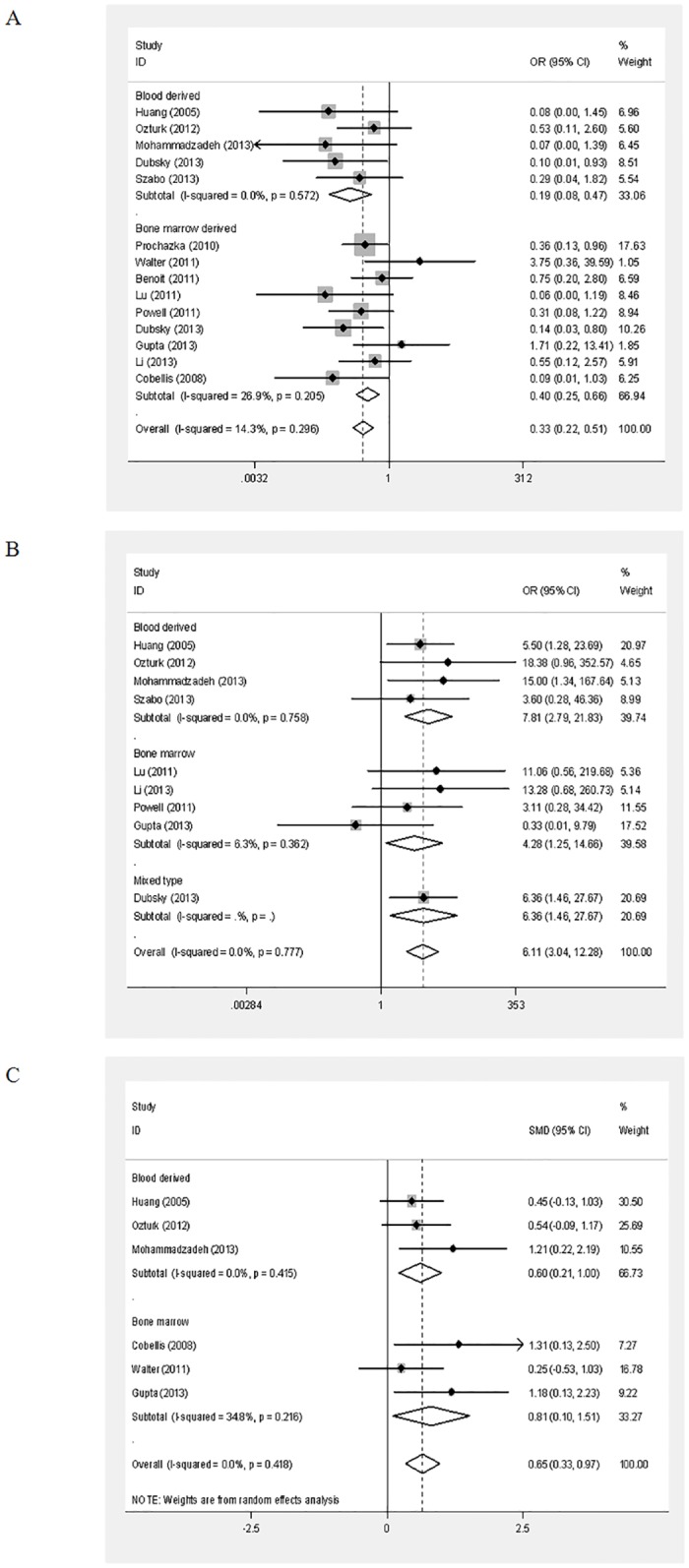
Forest plots of amputation (A), ulcer healing (B) and the ankle-brachial index (ABI) (C) usingcells from different sources. Amputation and ulcer healing were calculated using afixedeffects model, while ABI was calculated using a randomeffects model. CI:confidence interval; OR:odds ratio.

Significant improvementswere shown inamputation (OR = 0.33, 95%CI = 0.22–0.51; P<0.001), ulcer healing (OR = 6.11, 95%CI = 3.04–12.28; P<0.001), and ABI (SMD = 0.65, 95%CI = 0.33–0.97; P<0.001) in the stem cell-based therapy group compared with the controls (overall data in Fig 3). The subgroup analysis foundthat a significant decrease in amputations was found at 3months (OR = 0.32, 95%CI = 0.17–0.60; P<0.001), 6months (OR = 0.36, 95%CI = 0.20–0.67; P = 0.001) and 12months or longer (OR = 0.18, 95%CI = 0.04–0.77; P = 0.020) for the stem cell-based therapy group compared tothecontrols. A significant difference was also found inulcer healing at 3months (OR = 9.97, 95%CI = 3.53–28.22; P<0.001) and 6months (OR = 4.97, 95%CI = 1.85–13.30; P = 0.001), but not at 12months or longer for the stem cell-based therapy group compared tothecontrols(S3 Table). Moreover, a significant difference was found inABI at 3months (SMD = 0.53, 95%CI = 0.18–0.88; P = 0.003), 6months (SMD = 1.18, 95%CI = 0.13–2.23; P = 0.03), and 12months or longer for the stem cell-based therapy group compared tothecontrols (SMD = 1.32, 95%CI = 0.13–2.50; P = 0.028)(Fig 4 and S3 Table). In addition, the subgroup analysis based on the cell source showed that a significant improvement was found foramputation (Blood-derived: OR = 0.19, 95%CI = 0.08–0.47; P<0.001; Bone marrow-derived: OR = 0.40, 95%CI = 0.25–0.66; P<0.001), ulcer healing(Blood-derived: OR = 7.80, 95%CI = 2.79–21.83; P<0.001; Bone marrow-derived: OR = 4.28, 95%CI = 1.25–14.66; P = 0.021; Mixed type: OR = 6.36, 95%CI = 1.46–27.67; P = 0.014), and ABI (Blood-derived: SMD = 0.60, 95%CI = 0.21–1.00; P = 0.003; Bone marrow-derived: SMD = 0.74, 95%CI = 0.19–1.30; P = 0.009) in both the blood-derived andbone marrow-derived therapy groupscompared to the control group (Fig 3 and S4 Table).

The meta-analysis also showed that no significant difference was found inall-cause mortality(OR = 0.80, 95%CI = 0.39–1.64; P = 0.546) inthestem cell-based therapygroupcompared to the control group (Table 3).

**Table 3:** All-cause death at the longest available follow-up, as reported by the included studies and pooled using the Peto method.

Study	Death (Cell transplanted vs. Control, n/N)
**Huang et al**	0/14 vs. 0/14
**Cobellis et al**	0/10 vs. 0/9
**Prochazka et al**	5/42 vs. 8/54
**Benoit et al**	1/34 vs. 1/14
**Lu et al**	—
**Powell et al**	1/32 vs. 1/14
**Walter et al**	3/19 vs. 3/18
**Ozturk et al**	0/20 vs. 0/20
**Mohammadzadeh et al**	0/7 vs. 0/14
**Dubsky et al**	1/17 vs. 2/22
**Li et al**	2/29 vs. 2/29
**Gupta et al**	2/10 vs. 0/10
**Szabo et al**	0/10 vs. 2/10
OR (95% CI)*	0.802(0.393–1.641)
P value*	0.546

OR: odds ratio; CI: confident interval;

* the OR, 95%CI and p value were calculated using the Peto method.

### Publication bias

**Fig 5:**
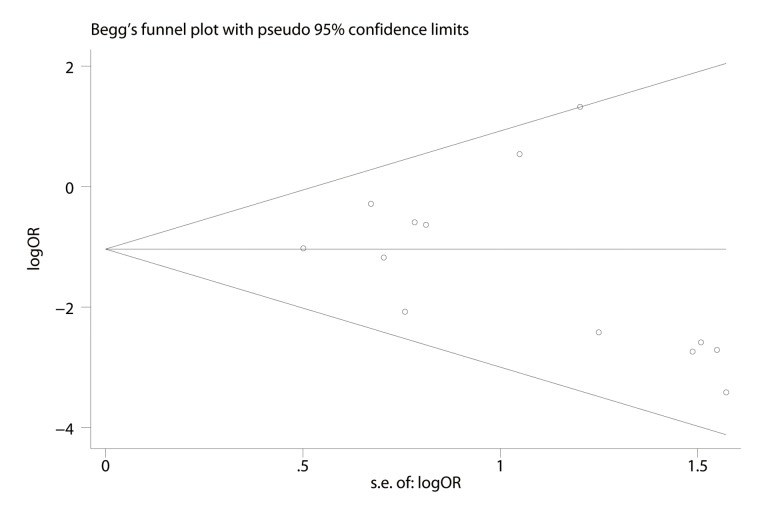
Begg’s funnel plot for amputation. logOR: logarithm of odds ratio; s.e.:standard error.

A Begg’s funnel plot for amputation showed that the studies were equally distributed around the overall estimate (Fig 5). Moreover, the Egger’s test showed no publication bias(p = 0.253).

## Discussion

Recent preclinical and clinical data have shown that autologous BMMNCs, BMMSCs and peripheral blood-derived proangiogenic cells might be promising therapeutic optionsforpatients with PAD or critical limb ischemia [22]. Several studies have demonstrated a significant improvementinABI, rest pain, transcutaneous oxygen pressure (TcPO_2_), amputation, and ulcer healing, with no significant impact on patient safety, after cell transplantation;however, inconsistent conclusions exist regarding the safety and efficacy of the clinical outcomes of cellular therapy because of the limited number of treated patients and variable procedures. In this study, we showed that a significant improvement inamputation, ulcer healing and ABI were found in the stem cell-based therapy group compared with the placebo control group, while no significant difference was found inthe all-cause mortality between the stem cell-based therapy and placebo control groups.

In this study, we selected amputation rate, ulcer healing and ABI as outcomes because they were most frequently presented in the studies and because the expression methodswerethesame among the studies. Thus, the data could be pooled together for analysis. Other outcomes, such as TcPO_2_, rest pain, walking distance, quality of life, vasculogenesis, or Fontaine score, were not employed due to differencesintheevaluation or expression methods. Moreover, all-cause mortality was employed because the safety evaluations using adverse events were incomplete in most of the studies.

Here, we also performed a subgroup analysis based on different follow-up times and cell sources. We used3months, 6months and 12months or longer as time points because most of the studiespresented data at these time points; we omitted the study conducted by Lasla *etal* [23] because they used 1-, 2-, and 4-month time points to displayresults. G-CSF-mobilized PBMCs were considered the stem cell-based therapy in this study because we hypothesizedthatthere wasa significant increasein the proportion of bone marrow-derived stem cells in the peripheral-derived blood mononuclear cells after G-CSF treatment [24]. In addition, BMMNCs and BMMSCs both represent a type of bone marrow stem cell; the difference is that BMMNCs may contain a small proportion of endothelial progenitor cells, which, according to experimental studies, could exert a significant effect on angiogenesis in PAD disease [25]. We did not perform a further subgroup analysis of studies using BMMNCs and BMMSCs due tothelimited number of studies with insufficient time points.

Fadini *et al* [26]previously performed a meta-analysis on autologous stem cell therapy for PAD. In their study, they included all the controlled and non-controlled, randomized and non-randomized trials using bone marrow or GM-CSF mobilized peripheral blood cells, and they concluded that autologous bone marrow therapy is a feasible, relatively safe and potentially effective therapeutic strategy for PAD patients. Although the cells in their study (they used bone marrow aspiration) were slightly different from thesein our study (we included BMMNCs and BMMSCs), the same property of these cells made us to conduct the same conclusions. Moreover, we thought we conducted a more accurate conclusion due to the exclusion of the non-controlled studies. Gao *et al* [27]reported a systemic review of autologous BMMNCs or G-CSF mobilized PBMCs to treat the PAD patients and they concluded that autologous hemopoietic stem cell transplantation may have positive effect on patients with PAD. In their study, they also selected uclear healing, limb salvage and ABI as the efficacy outcome. However, the included paper in their study on uclear healing, limb salvage and ABI were 5, 5 and 3, respectively, which were fewer in their study than the numbers in our study. Therefore, we may conducted a relative more reliable conclusion due to the more paper inclusion. Most recently, Wang *et al* published a meta-analysis studyonthe use of BMMNCs forthe treatment of patients with PAD [28]; they also selected ABI as the efficacy outcome, and performed a subgroup analysis based on the follow-up time. This groupconcluded that ABI was significantly increased at 12, 24 and 48 weeks but not at 4–8 weeks after cell-based therapy, which is consistent with our observations. However, a problem might arise because they also included studies that were published in Chinese and did not containplacebo control results.

In addition,our study contains some limitations. The first is the insufficient sample size that was used in our meta-analysis, especially given that the number of studies in some of the subgroup analyses was less than three. Moreover, a short-term follow-up time (3 and 6 months) was used in most of the included studies,the follow-up time of the trials was limited, and the long-term information regarding safety and efficacy wasscarce. Therefore, further analysis using a larger sample size and long-term clinical outcome indexes is required to achieve a more convincing conclusion.

In conclusion,assupported by ourmeta-analysis of 13 studies (275 stem cell-based therapy-treated patients and 252 control patients), our study suggests that stem cellseither from peripheral blood or blood marrow showcomparableshort-term, beneficial effects on patients with PAD. Although there are some limitations, our meta-analysis provides valuable information for the application of stem cell-based therapy in patients with PAD.

## Supporting Information

S1 Checklist PRISMA checklist. (DOCX)

S1 Diagram PRISMA flow chart. (DOCX)

S1 Table Search Strategy. (DOCX)

S2 Table Screening results of 49 papers. (DOCX)

S3 Table Effect of stem cell therapy over time (DOCX)

S4 Table Effect of stem cell thearpy with different source of cells. (DOCX)
