# Supplementary material for: Therapeutic Efficacy of Stem Cell-based Therapy in Peripheral Arterial Disease: A Meta-Analysis
Source: PLoS One. 2015 Apr 29;10(4):e0125032. doi: 10.1371/journal.pone.0125032 (PMC4414514; doi:10.1371/journal.pone.0125032)
Supplement: S1 Diagram — (DOCX) [file pone.0125032.s002.docx]

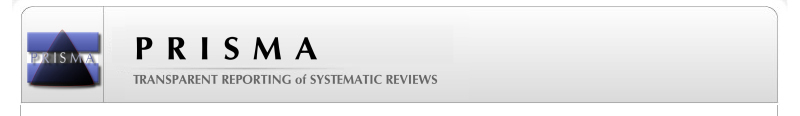
**PRISMA 2009 Flow Diagram**

Studies included in quantitative synthesis (meta-analysis)
(n =13 )

Studies included in qualitative synthesis
(n =13 )

Full-text articles excluded, with reasons
(n =3 )

Full-text articles assessed for eligibility
(n = 16 )

Records excluded
(n = 370 )

Records screened
(n =386 )

Records after duplicates removed
(n =55 )

Additional records identified through other sources
(n = 0 )

## Identification

## Eligibility

## Included

## Screening

Records identified through database searching
(n =441 )
