# Supplementary material for: Therapeutic Efficacy of Stem Cell-based Therapy in Peripheral Arterial Disease: A Meta-Analysis
Source: PLoS One. 2015 Apr 29;10(4):e0125032. doi: 10.1371/journal.pone.0125032 (PMC4414514; doi:10.1371/journal.pone.0125032)
Supplement: S1 Table — (DOCX) [file pone.0125032.s003.docx]

**S1 Table. Search Strategy (updated on 11/31/2014)**

| **Result:** |
| --- |
| 266 |
| **Translations:** |
| \| Peripheral artery disease \| "peripheral arterial disease"[MeSH Terms] OR ("peripheral"[All Fields] AND "arterial"[All Fields] AND "disease"[All Fields]) OR "peripheral arterial disease"[All Fields] OR ("peripheral"[All Fields] AND "artery"[All Fields] AND "disease"[All Fields]) OR "peripheral artery disease"[All Fields] \| \| --- \| --- \| \| Peripheral arterial disease \| "peripheral vascular diseases"[MeSH Terms] OR ("peripheral"[All Fields] AND "vascular"[All Fields] AND "diseases"[All Fields]) OR "peripheral vascular diseases"[All Fields] OR ("peripheral"[All Fields] AND "arterial"[All Fields] AND "disease"[All Fields]) OR "peripheral arterial disease"[All Fields] OR "peripheral arterial disease"[MeSH Terms] OR ("peripheral"[All Fields] AND "arterial"[All Fields] AND "disease"[All Fields]) \| \| PAD \| "Pathog Dis"[Journal] OR "pad"[All Fields] \| \| ischaemia \| "ischaemia"[All Fields] OR "ischemia"[MeSH Terms] OR "ischemia"[All Fields] \| \| limb \| "extremities"[MeSH Terms] OR "extremities"[All Fields] OR "limb"[All Fields] \| \| ischemia \| "ischaemia"[All Fields] OR "ischemia"[MeSH Terms] OR "ischemia"[All Fields] \| \| stem cells \| "stem cells"[MeSH Terms] OR ("stem"[All Fields] AND "cells"[All Fields]) OR "stem cells"[All Fields] \| \| bone marrow \| "bone marrow"[MeSH Terms] OR ("bone"[All Fields] AND "marrow"[All Fields]) OR "bone marrow"[All Fields] \| \| progenitor cells \| "stem cells"[MeSH Terms] OR ("stem"[All Fields] AND "cells"[All Fields]) OR "stem cells"[All Fields] OR ("progenitor"[All Fields] AND "cells"[All Fields]) OR "progenitor cells"[All Fields] \| \| cells \| "cells"[MeSH Terms] OR "cells"[All Fields] \| \| mesenchymal stem cells \| "mesenchymal stromal cells"[MeSH Terms] OR ("mesenchymal"[All Fields] AND "stromal"[All Fields] AND "cells"[All Fields]) OR "mesenchymal stromal cells"[All Fields] OR ("mesenchymal"[All Fields] AND "stem"[All Fields] AND "cells"[All Fields]) OR "mesenchymal stem cells"[All Fields] \| \| adipose tissue \| "adipose tissue"[MeSH Terms] OR ("adipose"[All Fields] AND "tissue"[All Fields]) OR "adipose tissue"[All Fields] \| \| stromal cells \| "stromal cells"[MeSH Terms] OR ("stromal"[All Fields] AND "cells"[All Fields]) OR "stromal cells"[All Fields] \| \| vascular \| "blood vessels"[MeSH Terms] OR ("blood"[All Fields] AND "vessels"[All Fields]) OR "blood vessels"[All Fields] OR "vascular"[All Fields] \| \| Humans[Mesh] \| "humans"[MeSH Terms] \| |
| **Database:** |
| PubMed |
| **User query:** |
| ((((((Peripheral artery disease))OR(Peripheral arterial disease))OR(PAD))OR(Claudication)OR(limb ischaemia))OR (limb ischemia)))AND((((((((stem cells))OR(bone marrow stem cells))OR(progenitor cells)) OR (mononuclear cells))OR((mesenchymal stem cells)OR mesenchymal stem-like stem cells))OR(adipose tissue derived regenerative cells))OR(vascular stromal cells))OR(vascular derived stem cells))AND((Clinical Trial[ptyp] OR Letter[ptyp] OR Controlled Clinical Trial[ptyp] OR Randomized Controlled Trial[ptyp]) AND "last 15 years"[PDat] AND Humans[Mesh]) |
