# Supplementary material for: Therapeutic Efficacy of Stem Cell-based Therapy in Peripheral Arterial Disease: A Meta-Analysis
Source: PLoS One. 2015 Apr 29;10(4):e0125032. doi: 10.1371/journal.pone.0125032 (PMC4414514; doi:10.1371/journal.pone.0125032)
Supplement: S2 Table — (DOCX) [file pone.0125032.s004.docx]

**S2 Table The screening results of 49 papers**

| Order number | Title [PMID] | Reason for exclusion |
| --- | --- | --- |
| 1 | Effect of autologous transplantation of bone marrow cells concentrated with the MarrowXpress system in patients with critical limb ischemia [23375329] | No control study |
| 2 | Intra-arterial allogeneic mesenchymal stem cells for critical limb ischemia are safe and efficacious: report of a phase I study[23307180] | No control study |
| 3 | A phase II trial of autologous transplantation of bone marrow stem cells for critical limb ischemia: results of the Naples and Pietra Ligure Evaluation of Stem Cells study[23197862] | No control study |
| 4 | Peripheral blood mono-nuclear cells implantation in patients with peripheral arterial disease: a pilot study for clinical and biochemical outcome of neoangiogenesis [23173612] | No control study |
| 5 | No difference in intra-arterial and intramuscular delivery of autologous bone marrow cells in patients with advanced critical limb ischemia [22472173] | No control study |
| 6 | Angiographic demonstration of neoangiogenesis after intra-arterial infusion of autologous bone marrow mononuclear cells in diabetic patients with critical limb ischemia [22289660] | No control study |
| 7 | Autologous stem cell therapy in the treatment of limb ischaemia induced chronic tissue ulcers of diabetic foot patients [22284892] | No control study |
| 8 | Controlled-release basic fibroblast growth factor for peripheral artery disease: comparison with autologous bone marrow-derived stem cell transfer [21810028] | bFGF therapy |
| 9 | Limb salvage using intramuscular injection of unfractionated autologous bone marrow mononuclear cells in critical limb ischemia: a prospective pilot clinical trial [21649569] | No control study |
| 10 | A randomized, controlled study of autologous therapy with bone marrow-derived aldehyde dehydrogenase bright cells in patients with critical limb ischemia [21594960] | No control study |
| 11 | Autologous bone marrow mononuclear cell therapy is safe and promotes amputation-free survival in patients with critical limb ischemia [21514773] | No control study |
| 12 | Combination stem cell therapy for the treatment of severe limb ischemia: safety and efficacy analysis [20498146] | No control study |
| 13 | Autologous bone marrow cell transplantation increases leg perfusion and reduces amputations in patients with advanced critical limb ischemia due to peripheral artery disease [19500466] | No control study |
| 14 | Long-term clinical outcome after intramuscular implantation of bone marrow mononuclear cells (Therapeutic Angiogenesis by Cell Transplantation [TACT] trial) in patients with chronic limb ischemia[19061721] | No control study |
| 15 | Autologous intra-arterial infusion of bone marrow mononuclear cells in patients with critical leg ischemia [18677289] | No control study |
| 16 | Bone marrow-derived mononuclear cell therapy induces distal angiogenesis after local injection in critical leg ischemia [18487998] | No control study |
| 17 | Treatment with autologous bone marrow mononuclear cells in patients with critical lower limb ischaemia. A pilot study [18450207] | No control study |
| 18 | Intramuscular or combined intramuscular/intra-arterial administration of bone marrow mononuclear cells: a clinical trial in patients with advanced limb ischemia [18212687] | No control study |
| 19 | Autologous mononuclear stem cell transplantation in patients with peripheral occlusive arterial disease [17293730] | No control study |
| 20 | Safety and efficacy of autologous progenitor cell transplantation for therapeutic angiogenesis in patients with critical limb ischemia [17251666] | No control study |
| 21 | Autologous bone-marrow mononuclear cell implantation in patients with severe lower limb ischaemia: a comparison of using blood cell separator and Ficoll density gradient centrifugation [16982058] | No control study |
| 22 | Therapeutical potential of blood-derived progenitor cells in patients with peripheral arterial occlusive disease and critical limb ischaemia [15855189] | No control study |
| 23 | Therapeutic angiogenesis by autologous bone marrow cell implantation for refractory chronic peripheral arterial disease using assessment of neovascularization by 99mTc-tetrofosmin (TF) perfusion scintigraphy [15468685] | No control study |
| 24 | Autologous bone-marrow mononuclear cell implantation improves endothelium-dependent vasodilation in patients with limb ischemia [15007007] | No control study |
| 25 | Therapeutic angiogenesis for patients with limb ischaemia by autologous transplantation of bone-marrow cells: a pilot study and a randomised controlled trial [12241713] | No control study |
| 26 | A randomized, controlled pilot study of autologous CD34+ cell therapy for critical limb ischemia [23192920] | CD34 therapy |
| 27 | Long-term clinical outcome after intramuscular transplantation of granulocyte colony stimulating factor-mobilized CD34 positive cells in patients with critical limb ischemia [22877866] | No control study |
| 28 | Waon therapy mobilizes CD34+ cells and improves peripheral arterial disease [20843662] | Waon therapy |
| 29 | Therapeutic vascular angiogenesis for intractable macroangiopathy-related digital ulcer in patients with systemic sclerosis: a pilot study24390937[uid] | No control study |
| 30 | Intraoperative adjunctive stem cell treatment in patients with critical limb ischemia using a novel point-of-care device [19896796] | Intraoperative stem cell treatment |
| 31 | Randomised comparison of G-CSF-mobilized peripheral blood mononuclear cells versus bone marrow-mononuclear cells for the treatment of patients with lower limb arteriosclerosis obliterans [18064333] | No control study |
| 32 | Local implantation of autologous mononuclear cells from bone marrow and peripheral blood for treatment of ischaemic digits in patients with connective tissue diseases [17309890] | No control study |
| 33 | Safety and efficacy of therapeutic angiogenesis as a novel treatment in patients with critical limb ischemia [20142004] | No control study |
| 34 | Early endothelial progenitor cells in bone marrow are a biomarker of cell therapy success in patients with critical limb ischemia [22040109] | EPC pathological analysis |
| 35 | Peripheral endothelial progenitor cells (CD133 +) for therapeutic vasculogenesis in a patient with critical limb ischemia. One year follow-up [17354105] | No control study |
| 36 | Effect of progenitor cell mobilization with granulocyte-macrophage colony-stimulating factor in patients with peripheral artery disease: a randomized clinical trial [24247554] | GM-CSF therapy |
| 37 | Intramuscular transplantation of G-CSF-mobilized CD34(+) cells in patients with critical limb ischemia: a phase I/IIa, multicenter, single-blinded, dose-escalation clinical trial [19711453] | No control study |
| 38 | Bone marrow mobilization with granulocyte macrophage colony-stimulating factor improves endothelial dysfunction and exercise capacity in patients with peripheral arterial disease [19540392] | GM-CSF therapy |
| 39 | Granulocyte colony-stimulating factor: a noninvasive regeneration therapy for treating atherosclerotic peripheral artery disease [16936417] | Bone marrow transplantation plus GM-CSF |
| 40 | Safety and efficacy of granulocyte-colony-stimulating factor administration following autologous intramuscular implantation of bone marrow mononuclear cells: a randomized controlled trial in patients with advanced lower limb ischemia [20078390] | No control study |
| 41 | Therapeutic angiogenesis in patients with severe limb ischemia by transplantation of a combination stem cell product [22079876] | No relevant follow-up study |
| 42 | Transplantation of autologous mononuclear bone marrow stem cells in patients with peripheral arterial disease (the TAM-PAD study) [17694378] | No relevant follow-up study |
| 43 | Therapy with autologous adipose-derived regenerative cells for the care of chronic ulcer of lower limbs in patients with peripheral arterial disease [23773718] | No relevant follow-up study |
| 44 | Autologous bone marrow mononuclear cell therapy improves symptoms in patients with end-stage peripheral arterial disease and reduces inflammation-associated parameters [24972744] | No control study |
| 45 | Adverse events during treatment of critical limb ischemia with autologous peripheral blood mononuclear cell implant [22330628] | No control study |
| 46 | Therapeutic angiogenesis in Buerger's disease (thromboangiitis obliterans) patients with critical limb ischemia by autologous transplantation of bone marrow mononuclear cells [19084740] | No control study |
| 47 | Transplantation of autologous bone marrow mononuclear cells for patients with lower limb ischemia [18706241] | No control study |
| 48 | Therapeutic angiogenesis of bone marrow mononuclear cells (MNCs) and peripheral blood MNCs: transplantation for ischemic hindlimb [18083329] | No control study |
| 49 | Phase I trial: the use of autologous cultured adipose-derived stroma/stem cells to treat patients with non-revascularizable critical limb ischemia [24438903] | No control study |

PMID: Pubmed-index
