# Supplementary material for: Therapeutic Efficacy of Stem Cell-based Therapy in Peripheral Arterial Disease: A Meta-Analysis
Source: PLoS One. 2015 Apr 29;10(4):e0125032. doi: 10.1371/journal.pone.0125032 (PMC4414514; doi:10.1371/journal.pone.0125032)
Supplement: S3 Table — (DOCX) [file pone.0125032.s005.docx]

**S3 Table. Effect of stem cell therapy over time**

|  | 95% CI | P value |
| --- | --- | --- |
| Amputation |  |  |
| 3-month | 0.32 ( 0.17, 0.60) | <0.001 |
| 6-month | 0.36 (0.20, 0.67) | 0.001 |
| 12-month or longer | 0.18(0.04, 0.77) | 0.020 |
| Ucler healing |  |  |
| 3-month | 9.97(3.53, 28.22) | <0.001 |
| 6-month | 4.97(1.85, 13.30) | 0.001 |
| 12-month or longer | 1.95 (0.47, 8.05) | 0.357 |
| ABI |  |  |
| 3-month | 0.53( 0.18, 0.88) | 0.003 |
| 6-month | 1.18(0.13, 2.23) | 0.03 |
| 12-month or longer | 1.32(0.13, 2.50) | 0.028 |

ABI: ankle-brachial index; CI: confidence interval
