# Supplementary material for: Therapeutic Efficacy of Stem Cell-based Therapy in Peripheral Arterial Disease: A Meta-Analysis
Source: PLoS One. 2015 Apr 29;10(4):e0125032. doi: 10.1371/journal.pone.0125032 (PMC4414514; doi:10.1371/journal.pone.0125032)
Supplement: S4 Table — (DOCX) [file pone.0125032.s006.docx]

**S4 Table. Effect of stem cell therapy with different source of cells**

|  | 95% CI | P value for Z |
| --- | --- | --- |
| Amputation |  |  |
| Blood derived | 0.192(0.08, 0.47) | <0.001 |
| Bone marrow derived | 0.40 (0.25, 0.66) | <0.001 |
| Ucler healing |  |  |
| Blood derived | 7.80 (2.79, 21.83) | <0.001 |
| Bone marrow derived | 4.28(1.25, 14.66) | 0.021 |
| Mixed type | 6.36 (1.46, 27.67) | 0.014 |
| ABI |  |  |
| Blood derived | 0.60 (0.21, 1.00) | 0.003 |
| Bone marrow derived | 0.74 (0.19, 1.30) | 0.009 |

ABI: ankle-brachial index; CI: confidence interval
